# Multimodal biomarkers and prediction models for stricturing and penetrating progression in Crohn's disease

**DOI:** 10.3389/fmed.2026.1958753

**Published:** 2026-09-30

**Authors:** Jingyao Yin, Ting Lei, Qingyi Zhao, Sijia Cao, Yanting Li, Chang Yin, Xiaoying Hu, Yin Shi

**Affiliations:** 1 Acupuncture Department, Seventh People’s Hospital of Shanghai University of Traditional Chinese Medicine, Shanghai, China; 2 Shanghai University of Traditional Chinese Medicine, Shanghai, China

**Keywords:** behavioral progression, crohn's disease, internal penetrating disease, multimodal prediction, precision medicine, prognostic biomarkers, risk stratification, stricturing progression

## Abstract

Crohn's disease can progress from non-stricturing, non-penetrating inflammation (B1) to stricturing (B2) or penetrating (B3) complications, yet risk is commonly recognized only after structural damage is established. This narrative review examines clinical, endoscopic, genomic, circulating, stool, tissue, imaging, radiomic, and computational biomarkers relevant to behavioral-progression risk and separates direct B1-to-B2 or source-defined B3 evidence from related prognostic and mechanistic evidence, while distinguishing internal penetrating from perianal fistulizing disease when source reporting permits. Structured searches of PubMed/MEDLINE, Embase, and Web of Science, supplemented by citation tracking, informed the selection of longitudinal human studies, prediction models, and mechanistic studies relevant to candidate feature selection. Ileal involvement, perianal fistulizing disease, persistent inflammatory burden, and endoscopic severity provide reproducible risk enrichment but insufficient individual-level precision. Genomic scores, plasma and stool proteins, tissue transcriptomics, shear-wave elastography, radiomics, and machine-learning models suggest potential value for multimodal risk stratification, but most lack independent external validation, calibration, transportability, and demonstrated incremental value over routinely available clinical variables. Direct prospective evidence is substantially more developed for B2 stricturing progression than for incident internal B3 penetrating progression; internal penetrating disease and perianal fistulizing disease are treated as distinct outcomes. Mechanistic studies suggest divergent fibrostenotic and tissue-destructive programs that provide biological guidance for biomarker selection. No biomarker or multimodal model identified in this review was ready to support routine biomarker-guided treatment escalation. Future studies should use well-characterized B1 baselines, prespecified structural outcomes, and prospective decision-impact testing that quantifies overtreatment.

## 1 Introduction

Crohn's disease now has objective monitoring, advanced therapy, and treat-to-target care, yet many patients still develop outcomes driven by structural bowel damage rather than inflammation alone: strictures, penetrating complications, abscesses, fistulas, hospitalization, surgery, and cumulative disability (1, 2). The Montreal behavioral categories carry clinical meaning: B1 identifies non-stricturing and non-penetrating inflammatory disease, B2 identifies stricturing disease, and B3 identifies penetrating disease at a specific assessment (3). Later B2 or B3 reclassification usually means that the intestine has crossed a structural threshold. The translational question is whether that transition can be predicted before irreversible damage is clinically established, as illustrated in Figure 1. For clinicians, the issue extends beyond detecting an established stricture or fistula. It is whether a newly diagnosed B1 patient should undergo closer transmural monitoring, molecular enrichment, or risk-adapted therapy before fixed narrowing, abscess, or fistula formation.

**Figure 1:**
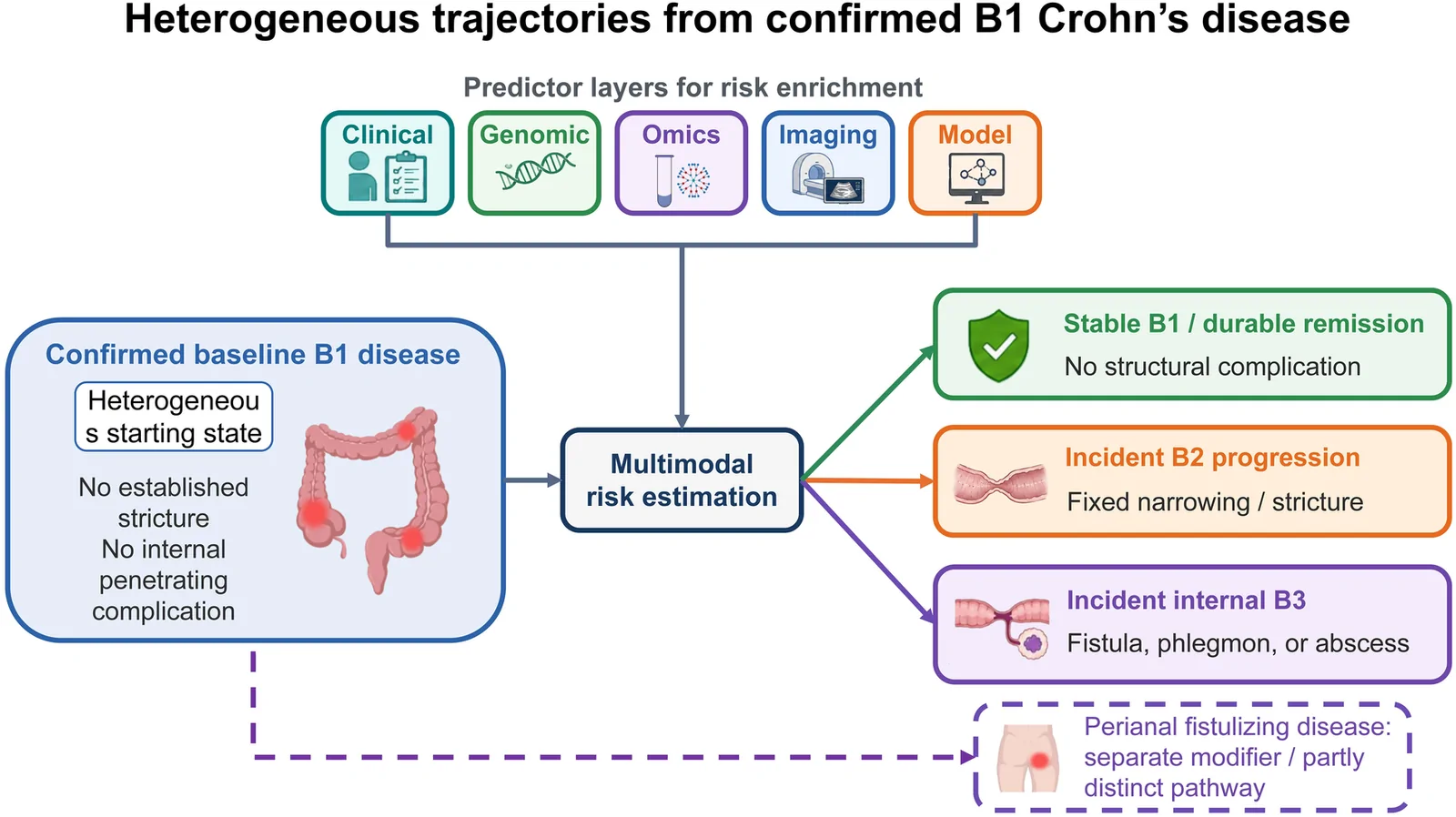
Heterogeneous trajectories from confirmed B1 Crohn's disease. Clinical, genomic, molecular, imaging, and computational predictor layers contribute to multimodal risk estimation in patients with objectively confirmed B1 disease. Outcomes include stable B1 disease or durable remission, incident B2 stricturing progression, and incident internal B3 penetrating progression. Perianal fistulizing disease is shown as a separate modifier and partly distinct pathway rather than as an interchangeable internal B3 outcome. The figure represents a risk-estimation framework, not a validated clinical prediction algorithm. Selected graphical elements were created with BioGDP.com; all remaining elements and the overall figure composition were created by the authors.

Earlier biomarker-oriented reviews framed Crohn's disease prognosis broadly, emphasizing clinical variables, inflammatory markers, serology, and genetics, whereas recent reviews of stricturing Crohn's disease have focused primarily on fibrosis biology, diagnosis, and management once structural narrowing is established (4–6). A recent phenotype-driven review of localized ileocecal Crohn's disease focused on inflammation-vs.-fibrosis phenotyping and treatment selection, with prospective prediction from confirmed B1 disease to incident B2 or internal B3 progression outside its scope (7). Since those syntheses, the evidence base has expanded through contemporary clinical and treatment-era cohorts (8–11), genomic and molecular approaches (12–15), imaging, radiomics, and elastography (16–18), single-cell and spatial studies of fibrostenotic tissue (19, 20), and longitudinal cohorts of internal fistulizing disease (21).

The present review addresses a narrower temporal and methodological question: what evidence supports prediction from an objectively characterized B1 state to incident B2 stricturing or internal B3 penetrating progression before structural complications are established? Four features distinguish this synthesis from broader severe-disease risk summaries and complication-centered reviews. First, direct prediction claims are restricted to longitudinal studies in which baseline phenotype and predictor timing permit assessment of incident progression; related prognostic and established-disease studies are labeled separately. Second, B2 stricturing, internal B3 penetrating, and perianal fistulizing disease are treated as distinct outcomes. Third, newer genomic, molecular, imaging, radiomic, and computational studies are organized using an author-derived evidence-directness framework that is explicitly separate from methodological quality. Fourth, model readiness is considered through discrimination, calibration, external validation, incremental value, and clinical net benefit. The translational aim is to identify which evidence is temporally appropriate for behavioral-progression prediction and what would be required for a model to become decision-ready.

## 2 Review approach and evidence-directness framework

### 2.1 Literature search strategy

PubMed/MEDLINE, Embase, and Web of Science Core Collection were searched from database inception through 31 July 2026. Search terms combined Crohn disease with behavioral or structural progression and prognostic biomarker or prediction concepts, including genomics, other omics, imaging, radiomics, elastography, machine learning, and artificial intelligence. Backward and forward citation tracking supplemented the database searches. Full database-specific strategies are provided in Supplementary Methods S1.

### 2.2 Eligibility criteria and study selection

Eligible primary studies addressed Crohn's disease behavioral progression, structural damage, prognostic biomarkers, or prediction models. Direct progression evidence required predictor measurement before the target outcome and a baseline phenotype permitting assessment of incident B2 stricturing and/or source-defined B3 progression. Studies with mixed or incompletely documented baseline behavior, or related outcomes such as surgery, bowel damage, hospitalization, treatment escalation, transmural damage, or composite severe-disease outcomes, were retained as indirect prognostic evidence. Established-complication, postoperative, tissue, organoid, and experimental studies were used for biological interpretation or candidate-feature selection. Preprints, conference abstracts, submitted manuscripts, personal communications, and reports without a relevant Crohn's disease progression, prognostic, or mechanistic question were excluded.

Study selection followed a single-reviewer workflow led by the lead reviewer using predefined eligibility and evidence-directness criteria. Records were assessed for topical relevance and, when needed, full-text eligibility, then classified by baseline phenotype, predictor-outcome temporality, endpoint definition, and role in the synthesis. Because the iterative narrative workflow did not prospectively maintain a deduplicated retrieval/exclusion log, initial retrieval and exclusion counts are unavailable. Supplementary Tables S1–S5 contain 52 unique reports in the detailed evidence map; at the report level, 3 were classified as Tier 1, 26 as Tier 2, and 23 as Tier 3.

### 2.3 Evidence appraisal

Evidence appraisal used an author-derived evidence-directness framework; a formal risk-of-bias or certainty-of-evidence instrument was not applied. Tier 1 required a confirmed B1 baseline, predictor measurement before outcome, longitudinal follow-up, and an incident B2 and/or source-defined B3 endpoint. Tier 2 comprised related prognostic evidence with mixed or incompletely documented baseline behavior or related structural/clinical outcomes. Tier 3 comprised established-complication or mechanistic evidence, including established strictures or fistulas, postoperative recurrence, tissue studies, organoids, or experimental models. The framework classifies proximity to incident behavioral progression from confirmed B1 disease, while methodological quality and clinical readiness are assessed separately. A Tier 1 study may therefore vary in methodological quality, while a rigorous Tier 3 study may provide strong mechanistic evidence despite lower directness to the progression question. Source-defined B3 endpoints retained the definitions used in the original studies; the term internal B3 was used only when internal penetrating events were explicitly distinguished from perianal disease in the source study. This framework guided Tables 1–3, Supplementary Tables S1–S5, and Figure 2.

**Figure 2:**
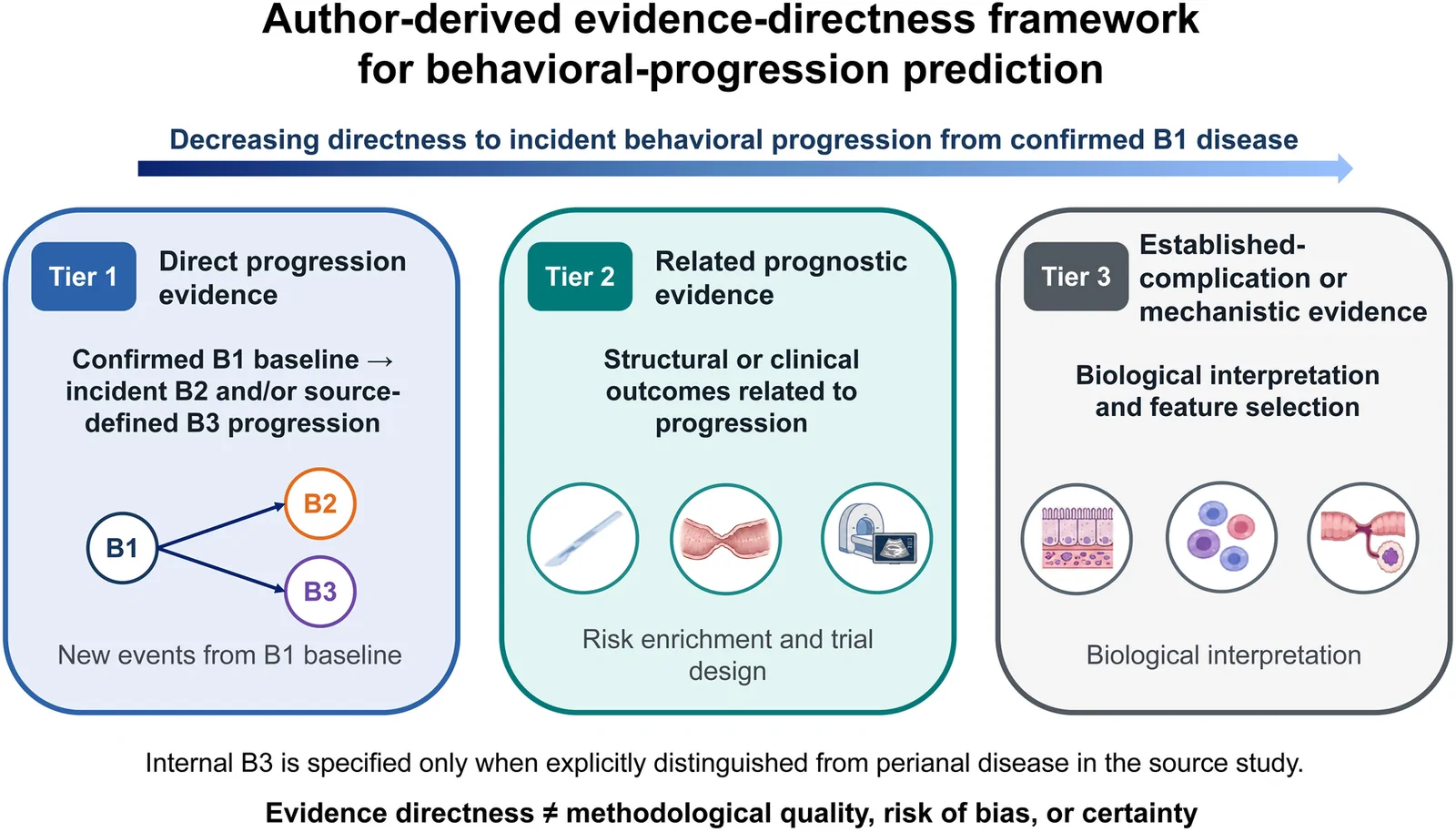
Author-derived evidence-directness framework for behavioral-progression prediction. Tier 1 evidence begins with confirmed B1 disease and directly evaluates incident B2 and/or source-defined B3 behavioral progression. The term internal B3 is used only when internal penetrating events are explicitly distinguished from perianal disease in the source study. Tier 2 comprises related prognostic evidence, including mixed-baseline or related structural and clinical outcomes. Tier 3 comprises established-complication or mechanistic evidence used for biological interpretation and feature selection. The tiers classify proximity to incident behavioral progression from confirmed B1 disease only; they are not a validated evidence-grading system and are separate from methodological quality, risk of bias, or certainty of evidence. Detailed classification is provided in Supplementary Tables S1–S5. Selected graphical elements were created with BioGDP.com; all remaining elements and the overall figure composition were created by the authors.

**Table 1:** Summary of prognostic-marker domains and current clinical readiness.

Predictor domain	Representative signals	Primary relevance	Evidence-directness tier	Validation status	Current use and principal limitation
Clinical and endoscopic	Ileal location; perianal fistulizing disease; persistent inflammation; endoscopic severity	Baseline enrichment and longitudinal reassessment for B2/internal-B3 risk; perianal fistulizing disease considered separately	Tier 1-2	Partially replicated; thresholds and treatment-era effects vary	Immediately available, but insufficient individual precision; should trigger reassessment rather than automatic escalation.
Genomic	*NOD2*/GenePy; genotype-derived transcriptomic scores	Stable enrichment, particularly for ileal and stricturing trajectories	Tier 1-2	Testing datasets exist, but ancestry and subgroup transportability remain limited	One-time testing is feasible; no validated treatment threshold or universal calibration.
Blood and stool	Plasma-protein panels; tissue inhibitor of metalloproteinases 2; collagen-turnover and neutrophil markers	Peripheral assessment of remodeling, innate immunity, and tissue injury	Tier 1-2	Mostly small or platform-specific cohorts; limited external validation	Promising for multimodal models; requires locked assays and incremental-value testing.
Tissue and multi-omics	Ileal wound-healing and matrix programs; microvillus phenotype; microbiome-metabolomics integration	Mechanistic anchoring and discovery of B2- and internal-B3-oriented candidate features	Tier 1-2	Sampling, batch effects, and spatial heterogeneity limit portability	Biologically informative but difficult to implement as routine tests.
Imaging	Shear-wave elastography; MRE; CTE; IUS; radiomics	Direct or related assessment of preclinical structural risk and established bowel damage	Tier 1-3	Among direct B1 studies, elastography is one of the clearest examples; many imaging studies begin after structural disease	Best positioned as structural confirmation after enrichment; acquisition and reader standardization are required.
Integrated models and artificial intelligence	Clinical, molecular, genomic, and imaging combinations	Absolute-risk estimation and linkage to monitoring or treatment decisions	Tier 1-3	Direct B1 models are mostly internally validated; some Tier 2/3 models report external validation, calibration, and decision curves.	Research use only; require absolute-risk calibration and demonstrated incremental net benefit over parsimonious benchmarks.

B1 non-stricturing, non-penetrating inflammatory behavior; B2, stricturing behavior; B3, penetrating behavior; CTE, computed tomography enterography; IUS, intestinal ultrasound; MRE, magnetic resonance enterography; *NOD2*, nucleotide-binding oligomerization domain-containing protein 2.

**Table 2:** Representative prediction and prognostic models with quantitative performance and validation characteristics.

Study or model	Baseline phenotype; N/events	Target outcome/horizon	Quantitative performance	Validation/incremental value	Evidence interpretation
Pediatric plasma-protein models (22)	Confirmed pediatric B1; *n* = 265; B2 events = 73, source-defined B3 events = 34.	Incident B2 and source-defined B3; mean event times 1,123 and 1,251 days.	B2 AUC 0.68 (95% CI 0.65–0.71) vs. clinical 0.52; B3 AUC 0.79 (0.76–0.82) vs. clinical 0.74.	Random-survival-forest internal/out-of-bag assessment; no independent external validation, calibration, or DCA reported.	Tier 1. Apparent incremental discrimination was larger for B2 than B3; decision impact remains untested.
Korean PrediXcan/TWAS model (15)	*n* = 430; 60 B2 and 73 B3 cases, but source case definitions included B2/B3 present at diagnosis; not a pure B1 cohort.	Source-defined B2/B3 status within 24 months; case groups included prevalent-at-diagnosis disease.	Clinical-only AUC 0.621 (B2) and 0.712 (B3); clinical+6 genes mean AUC 0.788 and 0.785 by LOOCV; sensitivity/specificity decreased vs. clinical-only.	No independent external validation, calibration, or DCA; ancestry-specific model.	Tier 2 for strict incident-prediction question. AUC gain did not translate into improved threshold sensitivity/specificity.
Shear-wave elastography nomogram (18)	Confirmed B1; *n* = 130; 27 (20.8%) progressed.	Incident source-defined composite B2/B3; median follow-up 33 months.	SWE HR 1.08 per kPa (95% CI 1.03–1.12); cutoff 12.75 kPa; nomogram AUC 0.792.	Retrospective single-cohort model; no independent external validation or externally calibrated decision threshold.	Tier 1. One of the most direct imaging examples, but event count was modest and B2/B3 were combined.
NOD2 GenePy model (23)	Mixed CD; *n* = 645; testing *n* = 161 (17/30 high-risk vs. 28/131 low-risk patients developed stricturing behavior).	Stricturing phenotype over longitudinal follow-up.	HR 2.092 overall; HR 3.164 in pediatric-onset disease; HR 4.89 for high-risk pediatric terminal-ileal subgroup vs. low-risk non-TI subgroup.	Training/testing split reported; no AUC, calibration, DCA, or external geographic validation reported.	Tier 2. Useful genomic enrichment with strong subgroup interaction, not confirmed-B1 prediction.
VAT radiomics model (16)	Mixed baseline behavior; *n* = 256; training 156/28 events, test 100/16 events.	Source-defined composite B2/B3 progression or Crohn's disease-related surgery.	Test AUC 0.850 (95% CI 0.764–0.913); VAT HR 9.285; VAT vs. SAT AUC 0.850 vs. 0.786; adding SAT to VAT: IDI 0.031, *P* = 0.102.	Multicenter test cohorts; calibration curves and DCA reported; no significant incremental gain from adding SAT.	Tier 2. Stronger validation than many models, but mixed baseline behavior and composite outcome limit directness.
Clinical model in established stricturing disease (24)	Established B2; *n* = 341 (internal 271/27 events; external 70/43 events).	Penetrating progression or surgery within 2 years.	LR AUC 0.861 internal and 0.752 external; RF 0.492/0.647; XGB 0.559/0.608.	Independent external validation; calibration and DCA reported for the LR nomogram.	Tier 3 for the target question. Parsimonious LR outperformed more complex ML in downstream B2 prognosis.

AUC, area under the receiver operating characteristic curve; B1, non-stricturing, non-penetrating inflammatory behavior; B2, stricturing behavior; B3, penetrating behavior; CI, confidence interval; DCA, decision-curve analysis; HR, hazard ratio; IDI, integrated discrimination improvement; kPa, kilopascal; LOOCV, leave-one-out cross-validation; *NOD2*, nucleotide-binding oligomerization domain-containing protein 2; RF, random forest; SAT, subcutaneous adipose tissue; SWE, shear-wave elastography; TWAS, transcriptome-wide association study; VAT, visceral adipose tissue; XGB, extreme gradient boosting.

**Table 3:** Minimum requirements for validation and clinical implementation.

Domain	Current problem	Minimum requirement	Potential clinical action	Go/no-go criterion
Baseline state	Occult B2/internal-B3 disease can be mislabeled as progression	Objective confirmation of B1 with ileocolonoscopy plus appropriate cross-sectional imaging to exclude occult B2/internal-B3 disease	Define the population eligible for prediction	Do not develop a progression model if baseline structural disease cannot be excluded.
Outcome definition	Composite outcomes mix distinct biology and health-system effects	Separate incident B2, internal B3, perianal fistulizing disease, surgery, and bowel-damage outcomes	Match risk estimates to a specific consequence	Proceed only when endpoint adjudication is reproducible and clinically meaningful.
Model development	High-dimensional features and few events promote overfitting	Prespecified clinical benchmark, adequate event count, locked features, transparent missing-data handling	Generate absolute risk over a fixed horizon	Reject models that do not improve calibration or utility beyond the benchmark.
External validation	Random splits do not establish transportability	Temporal, geographic, ancestry, age, and treatment-era validation with recalibration when required	Determine where the model can be used	Do not claim generalizability from internal validation alone.
Clinical utility	Discrimination does not define a useful decision	Calibration, threshold analysis, and decision-curve/net-benefit evaluation	Intensify imaging, shorten reassessment, or enroll in a prevention trial	Proceed only if the intended action has a favorable benefit-to-harm balance.
Treatment interaction	Prognostic risk does not identify treatment benefit	Prospective testing of prespecified model-by-treatment interaction	Select therapy only if differential benefit is demonstrated	Do not use a prognostic marker as a treatment-selection marker without interaction evidence.
Implementation	Assays and imaging pipelines may not reproduce across centers	Standard operating procedures, quality control, model accessibility, and monitoring for drift	Integrate into a defined clinical workflow	Implementation requires reproducible output, clear responsibility, and auditable updates.

The author-derived evidence-directness tiers classify proximity to incident behavioral progression from confirmed B1 disease. B2 and B3 endpoints are retained as reported by the source studies; the term internal B3 is used only when internal penetrating events were explicitly distinguished from perianal disease. The tiers do not represent methodological quality, risk of bias, or certainty of evidence. Perianal fistulizing disease is treated separately when identifiable. Detailed evidence is provided in Supplementary Tables S1–S5.

B1 non-stricturing, non-penetrating inflammatory behavior; B2, stricturing behavior; B3, penetrating behavior.

## 3 Defining behavioral progression and prediction endpoints

### 3.1 Confirmed B1 disease, occult damage, and outcome definition

Montreal B1 denotes non-stricturing and non-penetrating inflammatory Crohn's disease, B2 denotes stricturing disease, and B3 denotes penetrating disease. These labels change over time: behavior is assigned at a point in time while tissue injury accumulates longitudinally. Early B2 or B3 reclassification may reflect true biological progression, improved detection of occult damage, or both. This is particularly relevant when ileocolonoscopy is supplemented by magnetic resonance enterography (MRE), computed tomography enterography (CTE), or intestinal ultrasound (IUS), and when probable small-bowel stricturing is detected before classical upstream dilation is present (25). Here, the imaging definition characterizes early or probable structural change at baseline. Tandem ileocolonoscopy and cross-sectional imaging can further reveal stricturing, penetrating, or skipping complications beyond ileocolonoscopy alone (26).

Longitudinal studies show that B1 remains heterogeneous over time. Pediatric studies have reported increasing stricturing complications during follow-up and prognostic or prediction signatures for complicated behavior or resection among children initially diagnosed with B1 disease (27–30). Adult and mixed cohorts similarly document disease-behavior evolution, repeated surgery, and bowel-damage trajectories, including long-term outcomes in incidentally diagnosed or asymptomatic Crohn's disease (31–34). Cross-cohort rate comparisons require attention to referral pattern, diagnostic delay, imaging intensity, age at diagnosis, and therapeutic era because each factor changes event ascertainment.

The Lémann Index and disease-severity index help connect behavioral classification to structural damage. These tools help frame prevention trials and separate inflammatory activity from cumulative bowel damage, although routine decision use in B1 remains limited (35). Postoperative and surgery-focused studies reinforce this distinction while also showing how much endpoints vary: some address postoperative morbidity or durable remission, whereas others address patency testing, stricture-related surgery, or long-term adverse events (36–39). Endpoint heterogeneity favors trajectory framing over a single pooled incidence estimate.

Study endpoints vary. Some studies measure B2/B3 reclassification, whereas others use surgery, bowel damage, treatment escalation, hospitalization, radiological progression, or composite severe-disease outcomes. These endpoints represent overlapping but distinct constructs. Surgery is influenced by access, local practice, and patient preference. Bowel damage scores capture structural burden but require standardized imaging. Composite outcomes improve power but may combine stricturing, internal penetrating, perianal fistulizing, inflammatory, and treatment-response biology. Future studies should report incident B2, internal B3, and perianal fistulizing outcomes separately whenever event numbers permit.

Prediction has clinical meaning only when the time origin is explicit. A patient labeled B1 after ileocolonoscopy alone may already have unrecognized small-bowel narrowing or penetrating disease that is subsequently found by cross-sectional imaging; such cases represent baseline structural disease detected later. Reports should specify diagnostic delay, the interval between diagnosis and baseline sampling, the imaging used to exclude occult B2/internal-B3 disease, and the criteria for probable stricturing. Treatment and monitoring after baseline are also part of the causal pathway. Corticosteroid, immunomodulator, biologic, surgical, and imaging exposures should be reported as time-varying modifiers, because intensive treatment or surveillance can respectively alter or accelerate detection of the observed phenotype (26, 33, 35).

### 3.2 Internal penetrating progression and perianal fistulizing disease

Internal penetrating progression and perianal fistulizing disease should be analyzed separately. The Montreal classification records penetrating behavior as B3 and perianal disease as an additional modifier alongside luminal behavior (3). Internal fistulas, phlegmon, or abscesses arise from transmural bowel-wall penetration and are appropriate incident internal B3 outcomes, whereas perianal fistulas follow a partly distinct anatomic and immunobiologic pathway. Cohorts of internal fistulizing disease provide clinical characterization and outcome evidence for luminal B3 (21). Studies of established perianal fistulas contribute monitoring, mechanistic interpretation, and related prognostic context; direct internal B3 evidence requires a confirmed luminal B1 baseline and prospective adjudication of incident internal B3 events. Combining these outcomes may improve statistical power but can obscure trajectory-specific biomarkers and treatment effects.

## 4 Clinical and endoscopic prognostic markers

Classical predictors remain a reasonable starting point. Contemporary and historical data support disease location and age at diagnosis as baseline context variables (27, 31). Persistent inflammatory burden remains prognostic (40). Emerging collagen-turnover biomarkers illustrate how remodeling biology may be captured beyond classical variables (41). NOD2 provides genomic risk enrichment (12). Early treatment exposure functions most appropriately as an effect modifier (28, 29). Their relevance is established, while individual-level precision remains limited. They enrich baseline risk, but actionable thresholds generally require molecular or imaging confirmation.

Location and perianal fistulizing disease remain easy-to-interpret signals. Ileal involvement is biologically plausible for stricturing risk because it is a common site of transmural inflammation, microbial sensing abnormalities, and fibrostenotic remodeling. Population-based cohort studies have incorporated phenotypic, genetic, serological, and psychological characteristics into prognostic models of complicated Crohn's disease and surgery (42); these studies contribute related prognostic evidence. Perianal fistulizing disease may mark a distinct fistulizing pathway and is best modeled separately from future internal B3 progression (43–45). Endoscopic burden and persistent subclinical inflammation add another layer. Simple Endoscopic Score for Crohn's Disease thresholds and remission-depth measures have been associated with progression or phenotype stability (46, 47). Persistent fecal calprotectin elevation is associated with subsequent disease progression (40).

Clinical variables are more informative as trajectories than as fixed labels. A patient with ileal disease who achieves sustained biomarker normalization and deep bowel remission has a different structural-risk profile from a patient with persistent fecal calprotectin elevation and incomplete endoscopic or transmural response. Studies linking persistent subclinical inflammation, a higher Simple Endoscopic Score for Crohn's Disease, incomplete bowel remission, and absent transmural healing to progression suggest that cumulative inflammatory burden often tells us more than a single measurement (40, 46–48). Still, thresholds differ across cohorts and are influenced by treatment timing, sampling frequency, and imaging intensity. The markers are currently better suited to trigger reassessment or structural imaging than to mandate treatment escalation on their own.

Early effective therapy also changes how classical predictors should be interpreted. Observational pediatric data suggest that early anti-tumor necrosis factor therapy may reduce B1-to-B2 progression, and other early biologic cohorts support the concept that risk may be modifiable (28, 29). Reverse-translation studies linking a penetrating-fibrosis signature to treatment response further suggest that therapeutic response and progression biology may intersect (49). Together, these observations support modifiable risk; biomarker-selected escalation still requires prospective validation. Clinical and endoscopic markers are summarized in Table 1, with detailed extraction in Supplementary Table S1; they are readily accessible but have limited individual precision.

## 5 Molecular, genomic, and multi-omic biomarkers

### 5.1 Genomic predictors: *NOD2*, GenePy, polygenic scores, and TWAS

NOD2 remains the clearest genetic example. It enriches risk while leaving substantial outcome heterogeneity. NOD2-related risk enrichment is supported by cohort and genomic-score studies. A NOD2-derived GenePy approach shows the shift from binary genotyping to per-patient deleteriousness scoring (12, 23, 50). PrediXcan/TWAS work uses genetically predicted terminal-ileal expression to model source-defined B2/B3 status within 24 months (15). The model was developed in a Korean cohort, so its apparent discrimination remains ancestry-specific until transportability is tested in independent Asian, European, North American, and multiethnic datasets. Post-diagnosis behavioral prediction therefore requires direct testing against incident progression endpoints.

Genomic predictors are stable over time, can be measured once, and remain stable despite short-term inflammatory activity. Their clinical value, however, depends on incremental information beyond age, disease location, and baseline structural assessment. In the GenePy study, genomic risk was more informative in pediatric-onset and terminal-ileal subgroups, illustrating interaction between genotype and anatomical context and the need for subgroup-specific risk estimates (23). Likewise, a transcriptome-wide association model derived in one ancestry may require recalibration or feature re-estimation elsewhere (15). Future genomic studies should report ancestry composition, absolute-risk calibration, subgroup performance, and whether the genomic layer changes a monitoring or treatment decision (50).

### 5.2 Circulating and stool biomarkers beyond classical serology

Serum and fecal biomarkers are more informative when they move beyond generic inflammatory activity toward remodeling, immune-cell, or tissue-injury biology. A pediatric inception-cohort plasma-protein study is a Tier 1 example because baseline B1 samples were used to estimate risk of future B2 stricturing and source-defined B3 complications with distinct protein signatures (22). Stool tissue inhibitor of metalloproteinases 2, collagen-turnover markers, a serum marker of type XVI collagen formation, and neutrophil-related proteins suggest that remodeling and innate-immune signals can be measured outside tissue (41, 51, 52). The latter studies vary in baseline phenotype and endpoint directness and therefore contribute mainly risk-enrichment or mechanistic evidence. All require validation, assay standardization, and proof of incremental value over classical predictors.

Discovery and implementation answer different questions. Protein and stool studies can identify biologically plausible signatures in small, deeply phenotyped cohorts, but a clinical assay requires preanalytic stability, platform reproducibility, a locked algorithm, prespecified thresholds, and prospective performance in an independent population. The pediatric plasma-protein models showed different discrimination for future B2 and source-defined B3 events, whereas collagen-turnover and neutrophil markers remain closer to research-stage enrichment (22, 41, 51, 52). Detailed molecular evidence is provided in Supplementary Table S2.

### 5.3 Tissue transcriptomics and multi-omic signatures

Tissue transcriptomics sits close to the biology of progression, although it is harder to implement. Treatment-naive ileal programs involving macrophage, fibroblast, and extracellular matrix activation have been associated with future stricturing behavior (53). A short-microvillus phenotype has been linked to progression from inflammatory to complicated disease (30). Transcriptomic profiling of established stricturing mucosa provides supportive but less direct evidence (54). Integrated microbiome-metabolomics-MRE studies point toward convergent bowel-damage biology (32). Fecal metabolomics also links phenotype and location (55). Monocyte-derived amphiregulin provides an additional mechanistic bridge to fibrosis and remains a mechanistic candidate requiring prognostic validation (56). Patient-derived organoid work has also linked molecular Crohn's disease subtypes to real-world clinical phenotypes and outcomes, providing related translational evidence for molecular stratification (57). Peripheral biomarkers become more convincing when they are anchored to tissue cell types and pathways.

Tissue-based signatures are biologically informative but are limited by sampling burden, spatial heterogeneity, and assay standardization. Their clinical utility must be evaluated separately from biomarker discovery; the PRedicting Outcomes for Crohn's Disease Using a Molecular Biomarker (PROFILE) trial is considered in Section 9.3 (58). Applicability to older adults with small-bowel Crohn's disease also warrants dedicated study because comorbidity and treatment constraints are poorly represented in pediatric discovery cohorts (59).

Tissue signatures may be most valuable as mechanistic anchors, with routine implementation constrained by sampling location, inflammatory activity, prior therapy, and spatial heterogeneity (30, 53, 54). A practical translational pathway is therefore to reduce tissue programs to smaller blood or stool panels, or to identify imaging features that reflect the same biology. Multi-omic integration also requires strict separation of discovery and validation data, because the number of candidate features commonly exceeds the number of progression events. Batch effects, missing modalities, and cohort-specific processing pipelines can otherwise produce internally coherent signatures with limited clinical portability (54–56).

## 6 Imaging biomarkers and structural-risk markers

Imaging matters in progression research because Crohn's disease is transmural. Endoscopy assesses mucosal activity, whereas MRE, CTE, IUS, and elastography can detect bowel-wall thickening, narrowing, prestenotic dilation, stiffness, fistulas, abscesses, mesenteric changes, and transmural inflammation. One of the most direct imaging studies identified evaluated shear-wave elastography in confirmed B1 disease, in which baseline bowel stiffness was associated with subsequent source-defined composite B2/B3 progression (18). Combined fecal calprotectin and IUS evidence of transmural healing is associated with less bowel-damage progression, while interval MRE findings provide related long-term prognostic information (48, 60).

Most other cross-sectional imaging and radiomic studies contribute related prognostic or established-disease evidence. MRE and CTE findings have shown prognostic associations with surgery, bowel damage, or adverse outcomes (61, 62), and radiomic analyses can characterize inflammation, fibrosis, and smooth muscle thickening within established strictures (17, 63). Stricture length, disease duration, and obstructive symptoms have been associated with later intervention in established ileal stricturing disease (64), whereas visceral adipose tissue radiomics offers a candidate structural-risk signal that still requires acquisition harmonization and external validation (16, 65). CTE and ultrasound studies of symptomatic or established stricturing disease further inform surgery-free survival, histological fibrosis, surgery risk, and biologic response, with endpoints downstream of incident progression from confirmed B1 disease (66–69).

The shear-wave elastography study illustrates both the strengths and limitations of a relatively direct imaging-prognosis design. Among 130 patients with confirmed B1 disease, 27 progressed during a median follow-up of 33 months; higher baseline stiffness remained associated with the composite progression endpoint, and a 12.75-kPa threshold was incorporated into a nomogram with reported discrimination of 0.792 (18). The study began at the appropriate clinical time point and evaluated an actionable, noninvasive measure. Its retrospective design, modest event count, source-defined composite B2/B3 endpoint, and lack of external validation limit immediate clinical use; the 12.75 kPa threshold requires prospective external validation before routine treatment decisions.

Clinical translation is constrained by variation in definitions, acquisition protocols, segmentation, operator technique, and prediction horizons. Implementation of cumulative structural-damage measures such as the Lémann Index remains inconsistent (70). Studies of psoas-muscle diffusion, post-biologic MRE, and cross-sectional abnormalities in preclinical disease broaden the structural-risk landscape (71–73), while cross-sectional imaging reviews and IUS studies address fibrosis assessment, near-term resection, Doppler associations with surgery, and general prognosis (74–78). Elastography and molecular imaging remain investigational for fibrosis discrimination, and perioperative IUS mainly informs downstream care (79–81). Creeping fat adds imaging-biological context, whereas tissue-layer proteomics of established strictures links radiological phenotypes to bowel-wall biology (82, 83). Direct clinical use will require prospective confirmed-B1 cohorts, standardized B2 and internal B3 adjudication, separate handling of perianal fistulizing disease, and externally validated thresholds that change monitoring or treatment decisions.

Other imaging studies largely answer different questions. MRE, CTE, and radiomics can characterize established strictures, distinguish inflammatory from fibrotic or smooth muscle components, or show prognostic associations with surgery and adverse outcomes in patients who already have structural disease (61, 63, 64, 68). These studies are valuable for feature engineering and downstream management and arise from established structural disease. For clinical translation, serial imaging should use standardized acquisition, segment-level matching, prespecified definitions, and reproducibility testing across readers and machines. The immediate clinical role of imaging is most plausibly structural-risk confirmation after clinical or molecular enrichment (48, 74, 77, 78). Detailed imaging evidence is provided in Supplementary Table S3.

## 7 Integrated prediction models and artificial intelligence

### 7.1 Model development and validation

Prediction models combine clinical, molecular, tissue, and imaging variables across different time horizons. We assessed their target population and outcome, quantitative performance, validation, incremental value over simpler comparators, and decision usefulness when reported. Table 2 summarizes representative models, with fuller quantitative extraction in Supplementary Table S4.

### 7.2 Clinical utility, interpretability, and decision thresholds

Discrimination alone is insufficient for clinical use. A decision-ready model should estimate absolute risk over a prespecified horizon, be calibrated and externally validated, add value beyond a prespecified clinical benchmark, and show favorable net benefit at actionable thresholds. Among relatively direct B1 studies, pediatric plasma-protein models showed AUCs of 0.68 for B2 and 0.79 for source-defined B3, while a shear-wave elastography nomogram achieved an AUC of 0.792 in confirmed B1 disease (18, 22). Neither study established externally calibrated absolute-risk thresholds or prospective decision impact.

Related prognostic studies illustrate the importance of endpoint directness even when methodological performance is strong. A multicenter visceral-adipose radiomics model reported test-set discrimination, calibration, and decision-curve analysis; its mixed baseline population and composite endpoint place it in related prognostic evidence (16).

### 7.3 Model complexity and incremental clinical utility

Model complexity is clinically useful when it produces reproducible incremental performance. The Korean PrediXcan/TWAS study increased mean AUC after adding imputed-expression features, but sensitivity and specificity at selected cutoffs declined relative to clinical variables alone, validation was internal, and the case definitions included some patients already classified as B2 or B3 at diagnosis (15). These findings show that higher AUC should be interpreted alongside threshold performance, external validation, and transportability.

Downstream models provide complementary methodological examples. In established stricturing disease, logistic regression outperformed random forest and extreme gradient boosting in a multicenter study, whereas a separate CTE radiomics nomogram improved discrimination and reclassification over simpler comparators and reported calibration and decision-curve benefit (24, 84). These studies address established B2 populations and show how added complexity can be justified by reproducible incremental value beyond a simpler benchmark.

### 7.4 Reporting, external validation, and transportability

Transportability requires validation beyond the development setting. Models derived from pediatric inception cohorts, ancestry-specific genomic cohorts, tertiary imaging centers, or established stricturing populations should be tested in the population in which they are intended to be used (15, 16, 23, 85–87). Reports should clearly state the time origin, baseline phenotype, prediction horizon, event count, missing-data strategy, model specification, and uncertainty. Table 3 summarizes minimum reporting and validation requirements.

## 8 Mechanistic interpretation of progression biomarkers

### 8.1 Shared upstream signals: ileal susceptibility and persistent inflammatory injury

NOD2 links inherited microbial sensing with ileal tissue context. Cohort and genomic-score studies support NOD2-related risk enrichment, while mechanistic work connects microbial sensing, inflammatory myeloid activity, and stromal responses (12, 13, 23, 50). Persistent inflammatory activity may sustain epithelial stress and wound-healing programs, providing shared biological context for later structural injury (8, 16, 40).

### 8.2 Fibrostenotic progression toward B2

Mechanistic evidence for fibrostenosis comes mainly from established B2 tissue. Single-cell, spatial, proteomic, signaling, and mesenteric-fat studies collectively support a model of stromal activation, extracellular matrix accumulation, smooth muscle remodeling, mesenteric change, and increasing stiffness (19, 20, 56, 83, 88–96).

### 8.3 Internal penetrating progression toward B3

Mechanistic evidence linked specifically to incident internal B3 progression remains sparse. Studies of established fistulizing disease, especially perianal disease, support hypotheses involving barrier failure, matrix degradation, and destructive myeloid–stromal communication (44, 45, 97–105).

### 8.4 Mechanisms as feature-selection tools and evidence boundaries

The mechanistic literature supports partly divergent tissue programs: fibrostenotic B2 is characterized by stromal activation, matrix accumulation, smooth muscle remodeling, mesenteric change, stiffness, and fixed narrowing, whereas internal B3 is more closely associated with barrier failure, matrix degradation, invasive inflammatory cell–stromal communication, and tissue-destructive programs. Perianal fistulizing disease provides complementary but anatomically distinct information. These mechanisms guide candidate-feature selection and interpretation.

For B2, hypothesis-generating features include collagen turnover, fibroblast and smooth muscle programs, mesenteric-fat remodeling, and reproducible stiffness or radiomic measures (18–20, 41). For internal B3, candidate domains remain exploratory. Source-defined B3 protein signatures (22) and established fistulizing-disease studies (44, 45, 97) provide indirect candidate-feature evidence involving epithelial barrier injury, matrix degradation, invasive myeloid–stromal communication, and fistula-associated fibroblast states; these findings should not be interpreted as validated internal-B3 predictors.

Additional molecular, stromal, mesenteric, imaging, organoid, and fistula studies are summarized in Figure 3 and Supplementary Table S5 (106–123).

**Figure 3:**
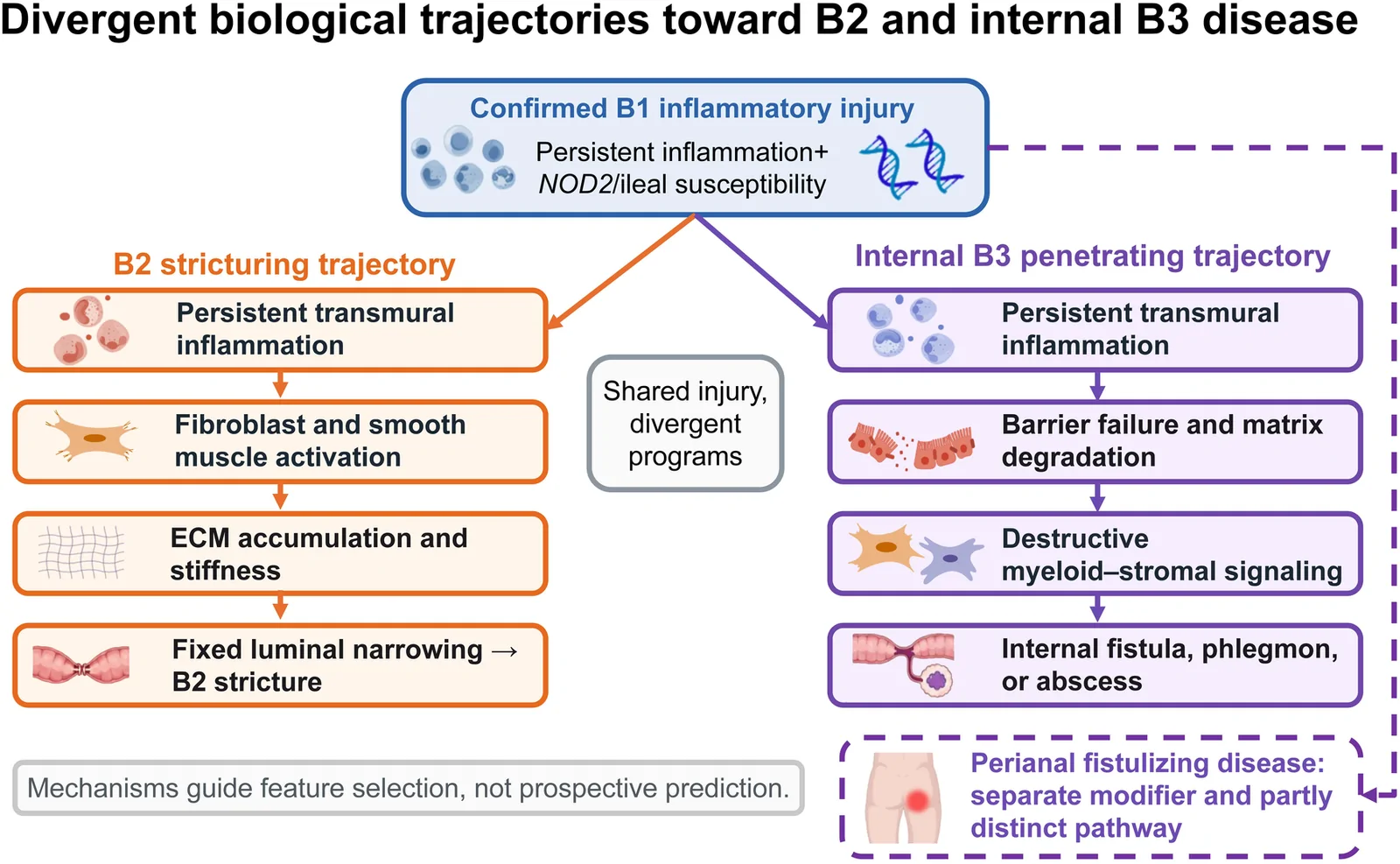
Divergent biological trajectories toward B2 and internal B3 disease. The proposed framework integrates mechanistic evidence suggesting that B2 and internal B3 may share upstream inflammatory injury but diverge in dominant tissue programs. The B2 trajectory emphasizes fibroblast and smooth muscle activation, extracellular matrix accumulation, tissue stiffness, and fixed luminal narrowing. The internal B3 trajectory emphasizes barrier failure, matrix degradation, destructive myeloid–stromal signaling, and internal fistula, phlegmon, or abscess formation. Perianal fistulizing disease is shown separately as a partly distinct pathway. These mechanisms are hypothesis-generating for biomarker selection and do not constitute direct prospective prediction evidence. Selected graphical elements were created with BioGDP.com; all remaining elements and the overall figure composition were created by the authors.

## 9 Discussion

### 9.1 Current clinical readiness and decision context

Within the literature identified in this review, current clinical, molecular, genomic, and imaging markers are best positioned for risk enrichment and for prioritizing closer monitoring, repeated intestinal ultrasound or MRE, and research-trial enrollment. Clinical and endoscopic variables are readily available but individually imprecise; genomics is stable but population-dependent; circulating and tissue signatures remain operationally immature; and imaging is closest to structural disease but often evaluates damage that is already present. The pediatric plasma-protein models and shear-wave elastography study are among the more direct examples identified; prospective validation of actionable escalation thresholds remains a priority (18, 22).

Clinical utility is action-dependent. A threshold used to intensify imaging can tolerate a different balance of sensitivity and specificity from one used to expose a patient to combination immunosuppression. Accordingly, discrimination should be interpreted alongside absolute risk, calibration, threshold-specific performance, external validation, incremental value over a prespecified clinical benchmark, and decision-curve or net-benefit analysis. Retention of more complex multimodal or machine-learning approaches should depend on reproducible improvement over simpler alternatives (16, 24). Table 1 summarizes the readiness of the major predictor domains.

### 9.2 Should B2 and internal B3 be predicted separately?

Stricturing and penetrating progression warrant separate outcome assessment. B2 has the more developed direct longitudinal evidence base, whereas direct prediction of incident internal B3 remains less mature; much of the available evidence is reported as source-defined B3 or composite B2/B3 outcomes (18, 20, 22). Internal penetrating complications and perianal fistulizing disease also differ in anatomy, ascertainment, and tissue biology; established perianal-fistula studies contribute related prognostic and mechanistic context (21, 43–45, 97–105).

A hierarchical strategy could first estimate the probability of any structural progression and then the conditional probability of B2 vs. internal B3, with perianal fistulizing disease modeled separately when relevant. Whether this approach outperforms parallel endpoint-specific models should be tested empirically.

### 9.3 Therapeutic prevention is distinct from behavioral-progression prediction: lessons from PROFILE

PROFILE evaluated therapeutic strategy and biomarker-guided treatment selection in newly diagnosed active Crohn's disease; it is considered here in that context. Sustained steroid-free and surgery-free remission through week 48 occurred in 149 of 189 patients (79%) assigned to top-down infliximab plus an immunomodulator, compared with 29 of 190 patients (15%) assigned to accelerated step-up treatment; however, the blood transcriptional classifier did not identify differential treatment benefit (58). The trial supports early effective therapy, while the classifier result did not demonstrate biomarker-directed differential treatment benefit.

The PROFILE findings illustrate a distinction that is important for future prevention trials. A prognostic model estimates the probability of an outcome under a given care pathway, whereas a treatment-predictive marker identifies differential benefit from one intervention vs. another. Observational pediatric studies suggest that early anti-tumor necrosis factor therapy may reduce B1-to-B2 progression, but confounding by indication and treatment era limit causal interpretation (28, 29). Any risk-guided treatment strategy should therefore be tested prospectively for benefit, toxicity, cost, and overtreatment.

### 9.4 Limitations of the current evidence

The available evidence is heterogeneous in ways that directly affect interpretation. Populations differ by age, ancestry, diagnostic delay, referral setting, treatment access, and surveillance intensity, while endpoints range from incident B2 or internal B3 behavior to perianal fistulizing disease, surgery, bowel damage, treatment escalation, and composite severe-disease outcomes. Baseline state is also inconsistent: some studies begin with objectively confirmed B1 disease, whereas others include occult damage, probable strictures, established stricturing disease, or postoperative recurrence. These differences limit direct comparison. Accordingly, downstream and mixed-phenotype cohorts are classified as related prognostic or contextual evidence.

Measurement and transportability introduce a second layer of limitation. Omics platforms, stool assays, radiomics pipelines, IUS protocols, MRE sequences, CTE reconstruction, and shear-wave elastography thresholds vary across centers. Performance may also shift across treatment eras and referral settings, because a predictor can partly encode access to advanced therapy, diagnostic delay, imaging intensity, or local thresholds for surgery in addition to biology. High-dimensional studies with modest event counts remain vulnerable to overfitting, and negative validation studies are less visible than positive discovery reports.

Publication and spectrum bias further reduce generalizability: tertiary cohorts contain more ileal, stricturing, penetrating, or treatment-refractory disease than community populations, which can inflate apparent performance in newly diagnosed B1 patients. The narrative design and single-reviewer screening may also introduce selection error and limit exhaustiveness. Postoperative recurrence and reprogression cohorts are classified as contextual evidence because their post-resection time origin is downstream of the target B1-to-B2/internal-B3 setting (124, 125). Likewise, a nomogram developed for stoma creation in patients already undergoing elective surgery for penetrating Crohn's disease addresses a downstream surgical decision in established penetrating disease (126).

### 9.5 Validation priorities and a staged research framework

Figure 4 presents a staged research validation pathway for progression-oriented risk stratification. Future studies should begin with objectively confirmed B1 disease, exclude occult B2/internal-B3 damage using appropriate cross-sectional imaging, prespecify a fixed prediction horizon, adjudicate incident B2 and internal B3 separately, record perianal fistulizing disease as a distinct modifier or endpoint, and treat post-baseline therapy as a time-varying exposure. Candidate models should then be compared with parsimonious clinical benchmarks and tested through temporal and geographic external validation, calibration assessment, and decision-curve analysis before prospective implementation studies. Central imaging review, blinded phenotype adjudication, and patient-centered structural outcomes would further improve reproducibility. Table 3 summarizes minimum development and validation requirements.

**Figure 4:**
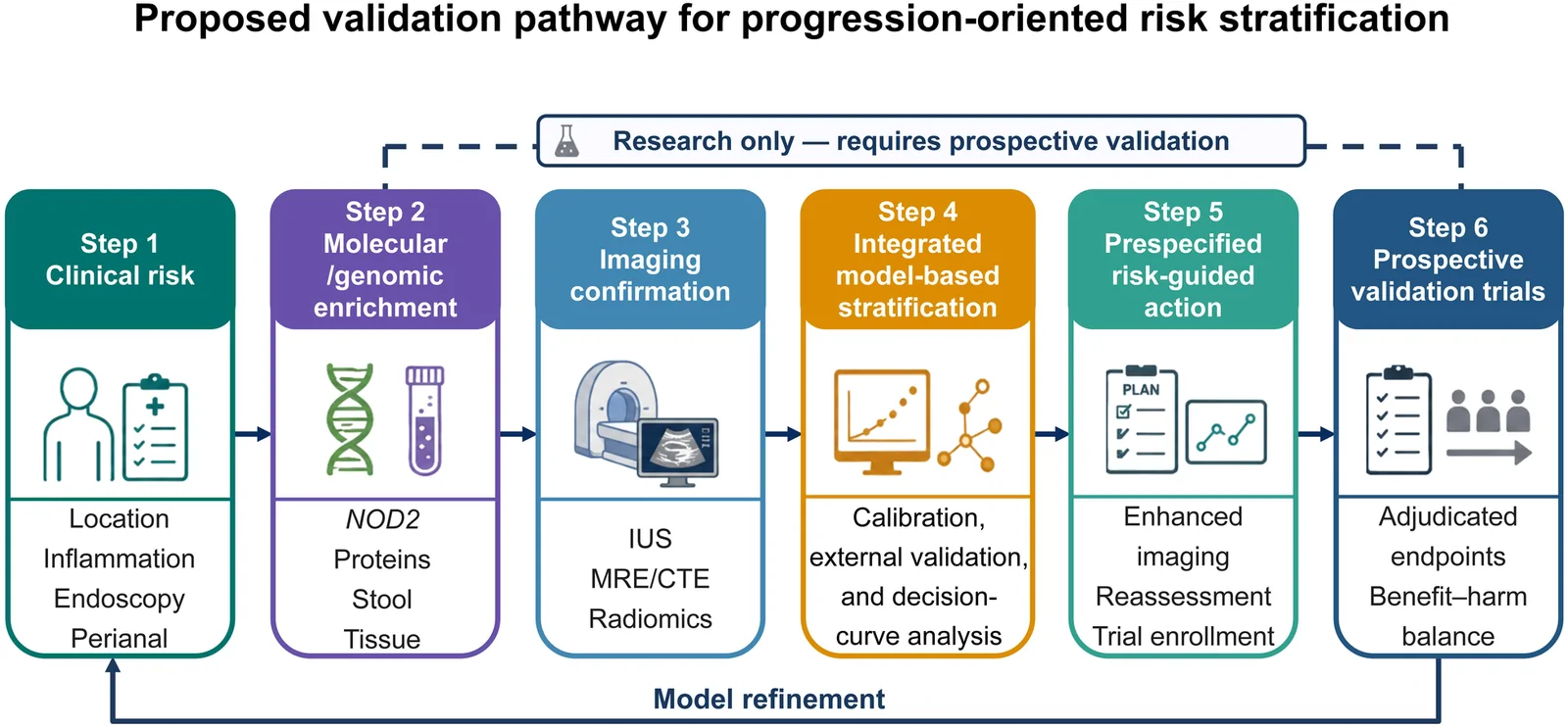
Proposed validation pathway for progression-oriented risk stratification. The sequence begins with baseline clinical assessment, adds molecular or genomic enrichment, confirms transmural or structural risk with imaging, tests an integrated model, links risk estimates to a prespecified risk-guided action, and evaluates that action in prospective validation and progression-prevention trials. Potential actions include enhanced imaging, shorter reassessment intervals, and trial enrollment; treatment escalation requires prospective evidence. The framework is a research agenda, not a validated clinical pathway. Selected graphical elements were created with BioGDP.com; all remaining elements and the overall figure composition were created by the authors.

Prospective trials should finally test whether risk-guided monitoring or therapy improves structural outcomes without unacceptable overtreatment. Lower-risk actions, such as earlier intestinal ultrasound or MRE, shorter reassessment intervals, or enrollment into disease-modification trials, may require less evidentiary certainty than automatic therapy escalation. Go/no-go criteria should include reproducible absolute-risk estimates, clinically meaningful net benefit, subgroup transportability, and evidence that the intervention can modify the outcome the model predicts.

## 10 Conclusion

Crohn's disease progression from B1 to B2 or internal B3 is heterogeneous, and perianal fistulizing disease warrants separate assessment. Across the literature identified here, current clinical, genomic, molecular, and imaging markers support risk enrichment; biomarker-guided treatment escalation still requires validated decision thresholds and prospective decision-impact evidence.

The field now needs prospective, well-phenotyped cohorts and decision-impact studies with emphasis on external validation and clinical utility. Mechanistic work can guide feature selection, whereas clinical translation depends on showing that a risk model changes monitoring or treatment in a way that improves structural outcomes without unacceptable overtreatment.
