# Supplementary material for: Multimodal biomarkers and prediction models for stricturing and penetrating progression in Crohn's disease
Source: Front Med (Lausanne). 2026 Sep 30;13:1958753. doi: 10.3389/fmed.2026.1958753 (PMC13662598; doi:10.3389/fmed.2026.1958753)
Supplement: Supplementary file 1 [file Supplementaryfile1.docx]

Supplementary Material

# Supplementary Table S1. Detailed clinical and endoscopic evidence relevant to behavioral progression in Crohn's disease.

| **Predictor or modifier** | **Representative evidence** | **Associated outcome / interpretation** | **Evidence-directness tier*** | **Validation status** | **Clinical use / readiness** | **Key limitation** |
| --- | --- | --- | --- | --- | --- | --- |
| Ileal/ileocolonic or extensive small-bowel location | Natural-history, anatomical-classification and phenotype/location metabolomic studies (31, 55, 86) | B2 or internal B3 progression where separately adjudicated, or related outcomes such as surgery, bowel damage, or severe disease course; useful as an anatomical enrichment feature. | Tier 1 when assessed in confirmed B1 cohorts; otherwise Tier 2 | Partially validated across natural-history cohorts, but definitions vary. | Routinely available; useful for baseline risk enrichment and selecting patients for molecular or imaging assessment. | Location definitions, imaging intensity and treatment era vary; risk may interact with *NOD2* and terminal-ileal biology. |
| Perianal fistulizing disease | Behavior-transition, perianal and fistulizing-disease cohorts (27, 43–45) | Perianal fistulizing burden and behavioral-transition context; not assumed to represent incident internal B3 progression. | Tier 2 for behavior-transition/prognostic cohorts; Tier 3 for established-fistula mechanistic studies; Tier 1 only when baseline luminal B1 and incident penetrating outcomes are prospectively adjudicated. | Partially replicated as a clinical enrichment feature, but evidence for incident internal B3 prediction is limited. | Routinely available; useful as a separate modifier and for determining whether perianal and internal penetrating outcomes should be modeled separately. | Perianal fistulizing disease follows a partly distinct pathway and should not be used as a surrogate for incident internal B3 progression. |
| Age at diagnosis / pediatric onset | Pediatric inception, registry, and complication-prognosis cohorts (27–30) | Complicated behavior, stricturing complications, penetrating complications, intestinal resection or severe disease course. | Tier 1/2 | Reproducible as a broad risk context, but effect size depends on cohort design and follow-up duration. | Routinely available; should be included as a baseline covariate in progression models. | Age effects are confounded by diagnostic era, pediatric monitoring intensity, treatment access and lifetime exposure window. |
| Persistent inflammatory biomarkers: CRP and fecal calprotectin | Subclinical inflammation and biomarker-monitoring studies (40, 46) | Composite progression, hospitalization, new fistula/abscess/stricture, treatment escalation or low-risk inflammatory control. | Mainly Tier 2; selected cohorts support Tier 1 interpretation | Partially validated; fecal calprotectin may outperform CRP in some settings. | Clinically available and inexpensive; useful for monitoring risk trajectory rather than as a stand-alone escalation trigger. | Inflammatory activity is prognostic but does not necessarily indicate irreversible structural damage; cut-offs and sampling frequency vary. |
| Endoscopic severity / incomplete mucosal remission | SES-CD and MM-SES-CD threshold studies, with supportive transmural-remission literature (8, 46, 48) | Disease progression, lower-risk remission state, bowel damage or phenotype progression. | Tier 1/2 for endoscopic progression studies; Tier 2 when transmural or bowel-damage endpoints are used | Partially validated; thresholds are increasingly interpretable but require external replication. | Clinically actionable as part of treat-to-target monitoring; should be integrated with cross-sectional imaging for structural risk. | Mucosal severity is not the same as transmural damage; escalation based on endoscopy alone may overtreat some B1 patients. |
| Classical serology panels: ASCA, anti-OmpC, anti-CBir1 and pANCA | Historical biomarker framework and pediatric/population prognostic models (4, 42, 85) | Complicated behavior, stricturing/penetrating phenotype, surgery or intestinal resection in selected cohorts. | Mainly Tier 2; selected pediatric models approach Tier 1 | Inconsistent across assays and cohorts; historically important but not sufficient alone. | May contribute to multi-variable models or first-pass enrichment, but should not be used as a stand-alone decision tool. | Assay variability, ancestry effects and limited decision thresholds reduce clinical readiness. |
| *NOD2*/CARD15 as a classical genetic risk marker | Long-term CARD15/*NOD2* cohorts and *NOD2*-derived genomic-risk studies (12, 23) | Ileal disease, stricturing tendency, and surgery risk; may be more informative when combined with terminal-ileal location or GenePy-type scoring. | Tier 2 for classical association studies; Tier 1/2 when tested in progression models | Partially validated, but population and phenotype dependent. | Stable baseline marker; best treated as genomic enrichment and discussed with modern GenePy/PRS approaches in Table 2. | Ethnicity, age at diagnosis, ileal involvement and variant weighting strongly modify performance. |
| Smoking, steroid exposure and hospitalization burden | Clinical severity and disease-severity index/risk-matrix models (35, 87) | Complications, surgery, bowel damage, treatment escalation or severe disease course. | Mainly Tier 2 | Partially validated as clinical-context variables, not specific progression biomarkers. | Routinely available; useful for contextual risk assessment, counseling and model adjustment. | Non-specific; strongly confounded by care access, disease severity, physician practice and treatment history. |
| Early effective therapy / treatment-era effect modifier | Pediatric anti-TNF, early biologic and therapeutic-era cohorts (28, 29, 49) | Reduced B1-to-B2 progression, modified complicated-course risk or changing behavior-progression trends across eras. | Tier 2 for observational treatment-exposure cohorts (28, 29); Tier 3 for reverse-translation mechanistic evidence (49). | Emerging and clinically plausible, but vulnerable to confounding by indication and treatment era. | Relevant to risk modification and trial design; should be modeled as treatment exposure or effect modifier, not as a classical predictor. | Observational treatment associations do not validate a routine biomarker-stratified escalation pathway. |

*The author-derived evidence-directness tiers classify proximity to incident behavioral progression from confirmed B1 disease. B2 and B3 endpoints are retained as reported by the source studies; the term internal B3 is used only when internal penetrating events were explicitly distinguished from perianal disease. The tiers do not represent methodological quality, risk of bias, or certainty of evidence. Perianal fistulizing disease is treated separately when identifiable.

Abbreviations: anti-CBir1, antibody to CBir1 flagellin; anti-OmpC, antibody to Escherichia coli outer membrane porin C; ASCA, anti-Saccharomyces cerevisiae antibodies; B1, non-stricturing and non-penetrating disease; B2, stricturing disease; B3, penetrating disease; CRP, C-reactive protein; NOD2, nucleotide-binding oligomerization domain-containing protein 2; pANCA, perinuclear anti-neutrophil cytoplasmic antibodies.

# Supplementary Table S2. Detailed molecular, genomic, and multi-omic evidence relevant to behavioral progression.

| **Predictor / signature** | **Biological layer and sample** | **Cohort / evidence type** | **Endpoint and horizon** | **Key performance or association** | **Evidence-directness tier*** | **Clinical readiness** | **Key limitation** | **Citation** |
| --- | --- | --- | --- | --- | --- | --- | --- | --- |
| *NOD2* GenePy genomic risk score | Genomics; blood/saliva DNA; per-patient *NOD2* deleteriousness metric | Mixed pediatric-adult CD cohort; derivation/testing design | Stricturing phenotype; longitudinal risk enrichment | High-risk *NOD2* group: HR 2.092; pediatric-onset subgroup: HR 3.164; high-risk *NOD2* plus terminal ileal disease: HR 4.89. | Tier 2 | Emerging risk-enrichment tool | Population, ancestry and disease-location dependence; does not define treatment threshold alone. | (23) |
| Korean-specific PrediXcan / TWAS model | Predicted transcriptomics; genotype-derived terminal-ileal expression | 430 Korean CD patients; multicenter retrospective modeling study | Source-defined B2/B3 status within 24 months; not pure incident progression | Reported AUC 0.788 for B2 and 0.785 for B3; internal-versus-perianal B3 classification was not assumed beyond the source definition. | Tier 1/2 | Research-stage model | Ancestry-specific model; external transportability beyond Korean cohorts remains uncertain. | (15) |
| RISK plasma protein panels | Proteomics; baseline plasma proteins measured using Olink platform | Pediatric B1 RISK inception cohort; case-cohort machine-learning analysis | Future B2 stricturing and source-defined B3 complications | Source-defined B3 5-protein model AUC 0.79; B2 4-protein model AUC 0.68. | Tier 1 | Promising but not decision-ready | Requires assay simplification, independent validation and proof of incremental value over clinical variables. | (22) |
| Baseline stool TIMP-2 | Fecal protein; matrix-inhibitor / tissue-remodeling pathway | 47 pediatric RISK patients with baseline stool samples | B2 stricturing and/or source-defined B3 progression as reported in the source study | Large association with progression reported; no validated B1-to-B2/B3 prediction threshold is established. | Tier 1 | Hypothesis-generating stool biomarker | Small sample; wide uncertainty likely; requires external validation and standardized stool assay. | (14) |
| Type I, III and IV collagen-turnover biomarkers | Serum ECM-remodeling biomarkers | Reported CD cohort; n = 37 | Presence and future progression of stricturing and penetrating CD | Associated with stricturing/penetrating disease and longitudinal progression; not a validated standalone B1-to-B2/internal-B3 prediction model. | Tier 1/2 | Research biomarker set | Small cohort; diagnostic versus prognostic contribution requires clearer separation. | (41) |
| PRO-C16: type XVI collagen formation marker | Serum ECM-formation marker | Reported CD cohort; n = 44 | Fibrostenotic strictures / fibrostenotic disease biology | Reflects intestinal fibrostenotic disease; no validated prospective B1-to-B2 prediction threshold reported. | Tier 2/3 | Mechanistic / diagnostic enrichment | More directly linked to established fibrostenosis than prospective B1 progression. | (52) |
| Serum neutrophil biomarkers | Serum innate-immune / neutrophil-associated proteins | Reported CD biomarker cohort; n = 100 | Disease progression and infliximab treatment outcomes | Prognostic performance was reported for mixed progression and treatment-response outcomes; used here as supportive risk-enrichment evidence. | Tier 2 | Research biomarker set | Outcome combines progression and treatment response; needs independent validation and decision-curve evidence. | (51) |
| Ileal ECM gene set combined with serology | Tissue transcriptomics plus serology; ileal biopsy gene-expression signature | Pediatric RISK ileal biopsy cohort | Future B2 stricturing behavior over 5 years | Model including ASCA, CBir1 and an eight-gene ECM set reported AUC 0.82. | Tier 1/2 | Biologically strong; implementation-limited | Requires ileal biopsy, standardized transcriptomic assay and reproducible clinical workflow. | (53) |
| Short ileal microvillus-length phenotype | Histology / tissue transcriptomics; ileal tissue phenotype | Pediatric RISK cohort with validation cohorts | Progression from inflammatory to complicated disease behavior | Shorter microvillus length associated with complicated-behavior progression and ECM, macrophage and fibroblast-enriched signatures. | Tier 1/2 | Research-stage tissue phenotype | No decision threshold for routine pathology; sampling and measurement standardization required. | (30) |

*The author-derived evidence-directness tiers classify proximity to incident behavioral progression from confirmed B1 disease. B2 and B3 endpoints are retained as reported by the source studies; the term internal B3 is used only when internal penetrating events were explicitly distinguished from perianal disease. The tiers do not represent methodological quality, risk of bias, or certainty of evidence. Perianal fistulizing disease is treated separately when identifiable.

Abbreviations: AUC, area under the receiver operating characteristic curve; B1, non-stricturing and non-penetrating disease; B2, stricturing disease; B3, penetrating disease; CI, confidence interval; CTE, computed tomography enterography; DCA, decision-curve analysis; DSI, Disease Severity Index; HR, hazard ratio; IDI, integrated discrimination improvement; LOOCV, leave-one-out cross-validation; NRI, net reclassification improvement; RF, random forest; RSF, random survival forest; SAT, subcutaneous adipose tissue; SWE, shear-wave elastography; TI, terminal ileal; TWAS, transcriptome-wide association study; VAT, visceral adipose tissue; XGB, extreme gradient boosting. NR denotes not reported.

# Supplementary Table S3. Detailed imaging and radiomic evidence relevant to behavioral progression.

| **Modality / marker** | **Representative study or feature** | **Cohort / context** | **Endpoint** | **Key result for this Review** | **Evidence-directness tier*** | **Interpretation and limitation** |
| --- | --- | --- | --- | --- | --- | --- |
| Intestinal ultrasound / shear-wave elastography (SWE) | Bowel stiffness assessed by SWE (18) | Confirmed B1 Crohn's disease; n = 130; 27/130 progressed; median follow-up 33 months | Incident source-defined composite B2/B3 disease-behavior progression | Bowel stiffness independently predicted progression: HR 1.08 per kPa (95% CI 1.03–1.12); proposed cut-off 12.75 kPa; nomogram AUC 0.792 | Tier 1 | One of the most direct imaging studies in this table; supports bowel stiffness as a candidate preclinical structural-risk marker, but the endpoint was composite. |
| CT-based visceral adipose tissue radiomics | Multicenter VAT radiomics signature (16) | Multicenter CTE/CT cohort of Crohn's disease with visceral adipose tissue radiomic extraction | Source-defined composite progression comprising stricturing/penetrating disease or Crohn's disease-related surgery | Radiomic signature showed good discrimination in validation/test cohorts, and decision-curve analyses suggested net benefit. | Tier 2 | Useful risk-enrichment evidence, but the mixed baseline population and related composite endpoint do not establish confirmed B1-to-B2/internal-B3 prediction. |
| Fecal calprotectin + intestinal ultrasound transmural healing | Transmural healing assessed by fecal calprotectin and IUS (48) | Crohn's disease monitoring cohort using biomarker plus ultrasound assessment | Bowel damage / transmural progression-related outcomes | Transmural healing was associated with substantially lower risk of adverse structural outcomes (HR 0.10 and 0.09 reported for separate endpoints) | Tier 2 | Supports monitoring structural response, but it is not a baseline B1 prediction study. |
| CTE radiomics of stricturing components | CTE radiomics model for inflammation, fibrosis and smooth muscle thickening (17) | Established stricturing Crohn's disease with imaging-pathology characterization | Inflammation, fibrosis and smooth muscle thickening in strictures | Radiomics was used to characterize tissue components in established stricturing disease; no incident B1 progression endpoint | Tier 3 | Valuable feature-selection and mechanistic evidence; should not be interpreted as prospective behavioral prediction. |
| MRE radiomics of fibrosis/inflammation | MRE radiomics to detect inflammation and fibrosis (63) | Crohn's disease with suspected or established stricturing/fibrotic bowel segments | Imaging discrimination of inflammation and fibrosis | Diagnostic/radiomic performance reported for fibrosis and inflammation; no direct baseline B1-to-B2/B3 prediction metric | Tier 3 | Useful for downstream fibrosis characterization and future feature engineering. |
| Longitudinal imaging / bowel-damage prediction | Predictors of bowel damage in long-term Crohn's disease progression (33) | Longitudinal Crohn's disease cohort with structural-damage outcomes | Bowel damage, surgery and complicated-disease outcomes | Identifies clinical/imaging factors associated with cumulative bowel damage rather than incident B1-to-B2/internal-B3 transition alone | Tier 2 | Appropriate for framing structural-damage trajectories; not for pooled incidence estimation. |
| MRE / microbiome / metabolomics integration | Microbiome and metabolomics integrated with MRE (32) | Crohn's disease cohort integrating stool/omics data with MRE-defined bowel damage | Bowel damage and imaging-defined structural burden | Supports imaging–molecular integration as a translational direction; no validated decision-ready progression model | Tier 2 | Conceptually important for multimodal risk stratification; requires prospective validation. |
| Stricturing-ileal imaging predictors of surgery | Imaging and clinical predictors of surgery or stricture-related outcomes (62, 64, 68) | Established or symptomatic stricturing ileal/small-bowel Crohn's disease cohorts | Surgery, stricture-related intervention or adverse stricturing outcomes | Risk associations were reported for surgery or stricture-related outcomes, but these cohorts do not start from confirmed B1 disease | Tier 2/3 | Useful for downstream management and endpoint definition; should not be rewritten as direct B1 progression prediction. |

*The author-derived evidence-directness tiers classify proximity to incident behavioral progression from confirmed B1 disease. B2 and B3 endpoints are retained as reported by the source studies; the term internal B3 is used only when internal penetrating events were explicitly distinguished from perianal disease. The tiers do not represent methodological quality, risk of bias, or certainty of evidence. Perianal fistulizing disease is treated separately when identifiable.

Abbreviations: AUC, area under the receiver operating characteristic curve; B1, non-stricturing and non-penetrating disease; B2, stricturing disease; B3, penetrating disease; CI, confidence interval; CT, computed tomography; CTE, computed tomography enterography; HR, hazard ratio; IUS, intestinal ultrasound; MRE, magnetic resonance enterography; SWE, shear-wave elastography; VAT, visceral adipose tissue.

# Supplementary Table S4. Quantitative performance, validation, and incremental-value characteristics of representative prediction and prognostic models.

| **Model / study** | **Baseline phenotype; N/events** | **Outcome / horizon** | **Effect size / discrimination** | **Calibration / validation / utility** | **Incremental-value or complexity finding** | **Evidence directness and use** |
| --- | --- | --- | --- | --- | --- | --- |
| RISK plasma-protein models (22) | Confirmed pediatric B1; n=265; B2=73 events, source-defined B3=34 events. | Incident B2 and source-defined B3; mean event times 1123/1251 days. | B2 AUC 0.68 (0.65-0.71), clinical 0.52, serology 0.62; B3 AUC 0.79 (0.76-0.82), clinical 0.74, serology 0.69. | Internal/out-of-bag RSF assessment; independent external validation, calibration, DCA, NRI/IDI not reported. | AUC gain vs clinical comparator: +0.16 for B2 and +0.05 for B3; decision impact not tested. | Tier 1; direct B1 molecular modeling with separate B2/source-defined B3 outcomes. |
| Korean PrediXcan/TWAS model (15) | n=430; controls 297 B1; B2=60 and B3=73 case groups included patients already B2/B3 at diagnosis. | Source-defined B2/B3 status within 24 months; not pure incident progression. | Clinical-only AUC 0.621/0.712 (B2/B3); clinical+6 genes mean AUC 0.788/0.785 by LOOCV. | No independent external validation, calibration, or DCA. At selected cutoffs, sensitivity/specificity decreased vs clinical-only models. | AUC increased by +0.167 (B2) and +0.073 (B3), but threshold performance did not improve. | Tier 2 for strict B1-to-incident-progression question; ancestry-specific and source definitions limit directness. |
| NOD2-derived GenePy model (23) | Mixed pediatric/adult CD; n=645; testing n=161; stricturing in 17/30 high-risk vs 28/131 low-risk. | Stricturing phenotype over longitudinal follow-up. | HR 2.092 overall; HR 3.164 pediatric; HR 4.89 in high-risk pediatric TI subgroup vs low-risk non-TI group. | Training/testing split; external geographic validation, AUC, calibration, DCA, NRI/IDI not reported. | Adding NOD2 risk group to anatomic context improved risk stratification, but no decision-threshold analysis was reported. | Tier 2; genomic risk enrichment rather than confirmed-B1 prediction. |
| Disease Severity Index validation (35) | Mixed behavior; n=300; 152 (50.7%) had complicated course. | Complicated CD course; median 5.1 months to event. | DSI AUC 0.66 (0.60-0.72) vs Lémann Index 0.56 (0.50-0.63); DSI>43 HR 2.18 (1.54-3.09). | Single-center validation; calibration, DCA and external validation not reported. | DSI discriminated better than Lémann Index (P=0.007), but endpoint and baseline phenotype were mixed. | Tier 2; related structural/clinical prognosis, not pure B1 progression. |
| Clinical model in established stricturing disease (24) | Established B2; n=341: internal 271 (27 events), external 70 (43 events). | Penetrating progression or surgery within 2 years. | LR AUC 0.768 training, 0.861 internal validation, 0.752 external; RF 0.908/0.492/0.647; XGB 0.785/0.559/0.608. | Independent external validation; calibration and DCA reported for LR nomogram. | LR outperformed RF/XGB in internal and external validation, showing that higher algorithmic complexity did not improve generalization. | Tier 3 for B1 target question; useful downstream methodological comparator. |
| VAT radiomics model (16) | Mixed baseline CD; n=256: training 156/28 events; test 100/16 events. | Source-defined composite B2/B3 progression or Crohn's disease-related surgery. | Test AUC 0.850 (0.764-0.913); VAT HR 9.285; SAT AUC 0.786. | Multicenter test cohorts; calibration curves and DCA reported. | Adding SAT to VAT did not significantly improve performance: IDI 0.031, P=0.102; VAT had greater net benefit than SAT. | Tier 2; quantitatively well-reported related prognostic model, but not confirmed-B1 progression. |
| SWE progression nomogram (18) | Confirmed B1; n=130; 27 (20.8%) progressed. | Incident source-defined composite B2/B3; median follow-up 33 months. | SWE HR 1.08/kPa (1.03-1.12); cutoff 12.75 kPa; nomogram AUC 0.792. | Retrospective single cohort; independent external validation and externally calibrated thresholds not reported. | Multivariable nomogram provided discrimination beyond single measurements, but incremental net benefit requires prospective validation. | Tier 1; direct imaging example with modest event count and composite endpoint. |
| CTE radiomics nomogram in established stricturing disease (84) | Established B2; n=219: train 153/50 events, test 66/22 events. | Clinical adverse outcomes at 1, 2 and 3 years. | Test C-index: clinical 0.752, radiomics 0.775, nomogram 0.849; nomogram AUC 0.874/0.863/0.956 at 1/2/3 years. | Calibration curves and DCA reported; test NRI 0.557 and IDI 0.206 vs clinical model. | Integrated nomogram improved discrimination/reclassification and net benefit over clinical model, but within established B2. | Tier 3 for B1 target question; useful example of how incremental value can be demonstrated. |
| Penetrating-disease stoma nomogram (126) | Established penetrating CD undergoing elective surgery; n=540 (train 405, validation 135); event count not reported in abstract. | Stoma creation at elective resection; downstream surgical decision. | Nomogram AUC 0.821; R² 0.394. | Internal validation with discrimination, calibration and clinical-usefulness assessment reported; no upstream B1 validation. | Model addresses a treatment/surgical decision in established penetrating disease rather than behavioral progression. | Tier 3; downstream B3 management only, not B1-to-internal-B3 prediction. |

*The author-derived evidence-directness tiers classify proximity to incident behavioral progression from confirmed B1 disease. B2 and B3 endpoints are retained as reported by the source studies; the term internal B3 is used only when internal penetrating events were explicitly distinguished from perianal disease. The tiers do not represent methodological quality, risk of bias, or certainty of evidence. Perianal fistulizing disease is treated separately when identifiable.

Abbreviations: AUC, area under the receiver operating characteristic curve; B1, non-stricturing and non-penetrating disease; B2, stricturing disease; B3, penetrating disease; CI, confidence interval; CTE, computed tomography enterography; DCA, decision-curve analysis; DSI, Disease Severity Index; HR, hazard ratio; IDI, integrated discrimination improvement; LOOCV, leave-one-out cross-validation; NRI, net reclassification improvement; RF, random forest; RSF, random survival forest; SAT, subcutaneous adipose tissue; SWE, shear-wave elastography; TI, terminal ileal; TWAS, transcriptome-wide association study; VAT, visceral adipose tissue; XGB, extreme gradient boosting. NR denotes not reported.

# Supplementary Table S5. Detailed predictor-mechanism-trajectory mapping in behavioral progression.

| **Predictor/mechanism layer** | **Representative evidence** | **Mechanistic pathway** | **Likely trajectory** | **Evidence-directness tier*** | **Use in the Review** | **Key limitation** |
| --- | --- | --- | --- | --- | --- | --- |
| Genetic susceptibility and ileal context | *NOD2*/CARD15, *NOD2*-derived risk and *NOD2*-driven myeloid–stromal niche studies (12, 13, 23). | Microbial sensing defects converge with ileal inflammation, myeloid activation and stromal remodeling. | Both; often B2-enriched with ileal disease | Tier 2–3 | Risk-enrichment and biological interpretation layer. | Modified by ancestry, age and disease location; not a deterministic clinical test. |
| Persistent inflammatory burden | Endoscopic severity, fecal calprotectin/CRP, persistent subclinical inflammation and remission-depth studies (8, 40, 46, 48). | Repeated injury may maintain epithelial stress, macrophage activation and wound-healing programs. | Both | Tier 1–2 | Links inflammatory control with structural-prevention rationale. | Activity markers are associated with complications inconsistently unless coupled to remodeling susceptibility. |
| Blood protein signatures separating B2 and source-defined B3 outcomes | Pediatric RISK plasma-protein panels estimated later stricturing and penetrating complication risk with distinct signatures (22). | B2 markers are more stromal/remodeling-linked; source-defined B3 markers point toward immune and tissue-invasive biology. | Both, but separable | Tier 1 | Direct support for reporting B2 and B3 outcomes separately, while retaining the source study’s B3 definition. | Needs external validation, assay simplification and incremental-value testing. |
| Matrix-remodeling biomarkers | Stool TIMP-2, collagen-turnover markers and PRO-C16 (14, 41, 52). | TIMP/MMP balance, collagen turnover and ECM formation provide measurable remodeling readouts. | Mainly B2; possibly complicated disease broadly | Tier 1–3 | Bridge between peripheral/stool biomarkers and fibrostenotic biology. | May be diagnostic for fibrosis rather than prospectively predictive; validation remains limited. |
| Ileal tissue and transcriptomic programs | Ileal transcriptomic and histological studies report macrophage/fibroblast activation, ECM programs and short microvillus phenotype (30, 53, 54). | Early ileal tissue may encode stromal activation, wound healing and epithelial remodeling before complications emerge. | Mainly B2; some shared signals | Tier 1–3 | Tissue anchor for blood, stool and imaging biomarkers. | Requires ileal sampling; spatial heterogeneity and assay standardization limit implementation. |
| Fibroblast heterogeneity and fibrosis-associated spatial networks | Single-cell and spatial transcriptomic studies of stricturing Crohn’s disease (19, 20). | Inflammatory fibroblasts, ECM-producing stromal cells and immune–stromal modules organise fibrostenotic tissue. | B2 | Tier 3 | Mechanistic explanation for B2-oriented predictors and feature selection. | Established/resection tissue; not validated as early B1 screening. |
| Smooth muscle, creeping fat and mechanical stiffness | SWE, VAT radiomics, delta-radiomics and creeping-fat/stromal studies (16, 18, 65, 96). | Fibroblast–smooth muscle–mesenteric fat crosstalk may produce stiffness, narrowing and radiomic remodeling signals. | Mainly B2 | Tier 1–3 | Connects molecular remodeling with imaging-confirmed structural risk. | SWE provides direct B1 evidence; many radiomic/fat studies address related outcomes or established disease rather than incident B1 progression. |
| Barrier disruption and fistula niches | Perianal fistula single-cell, spatial and imaging-mass-cytometry studies (97–99). | Barrier failure, myeloid–stromal crosstalk and destructive stromal phenotypes organise fistula tracts. | Established fistulizing disease; mechanistic relevance to internal B3 is indirect | Tier 3 | Mechanistic context for fistulizing biology; does not establish prediction of incident internal B3 progression. | Established fistula tissue, largely perianal; perianal and internal penetrating disease should not be treated as interchangeable. |
| Immune-remodeling programs in fistulizing disease | Immune profiling, TL1A-associated rectal remodeling and rectal organoid studies (45, 102, 103). | Th1/17 activation, epithelial injury, EMT-like programs and matrix degradation may drive tissue invasion. | Established fistulizing disease; potential relevance to internal B3 is indirect | Tier 3 | Links therapeutic targets and mucosal biology with fistulizing mechanisms; not direct internal-B3 prediction evidence. | Target plausibility and established-fistula biology do not prove prediction or prevention of incident internal B3 events. |
| Integrated B2 versus penetrating/fistulizing trajectory separation | Across studies reporting B2, source-defined B3/composite B2/B3, and fistula-spatial phenotypes, partially separable patterns are suggested (18, 20, 22, 97). | Shared inflammation may diverge toward fibrostenotic remodeling for B2 or tissue-invasive programs relevant to internal B3; perianal fistulizing evidence remains indirect for the latter. | Both, biologically separable; internal B3 evidence is less direct | Synthesis of Tiers 1–3 | Supports separate B2, internal B3, and perianal reporting and cautious use of composite endpoints. | Interpretive synthesis; prospective multimodal models must test whether separation improves decisions. |

*The author-derived evidence-directness tiers classify proximity to incident behavioral progression from confirmed B1 disease. B2 and B3 endpoints are retained as reported by the source studies; the term internal B3 is used only when internal penetrating events were explicitly distinguished from perianal disease. The tiers do not represent methodological quality, risk of bias, or certainty of evidence. Perianal fistulizing disease is treated separately when identifiable.

Abbreviations: B1, non-stricturing and non-penetrating disease; B2, stricturing disease; B3, penetrating disease; CRP, C-reactive protein; ECM, extracellular matrix; EMT, epithelial-to-mesenchymal transition; MMP, matrix metalloproteinase; MRE, magnetic resonance enterography; NOD2, nucleotide-binding oligomerization domain-containing protein 2; SWE, shear-wave elastography; TIMP, tissue inhibitor of metalloproteinases; TL1A, tumor necrosis factor-like cytokine 1A; VAT, visceral adipose tissue.

# Supplementary Methods S1. Literature search strategy and study-selection documentation.

This review used a structured narrative search with a literature cutoff of 31 July 2026. The database-specific strategies below document the search concepts and syntax used for PubMed/MEDLINE, Embase, and Web of Science Core Collection and support reproducibility of the search process.

**PubMed/MEDLINE**

("Crohn Disease"[Mesh] OR Crohn*[tiab]) AND ("Disease Progression"[Mesh] OR progress*[tiab] OR "disease behavio*"[tiab] OR B1[tiab] OR B2[tiab] OR B3[tiab] OR nonstrictur*[tiab] OR "non-strictur*"[tiab] OR nonpenetrat*[tiab] OR "non-penetrat*"[tiab] OR strictur*[tiab] OR stenos*[tiab] OR penetrat*[tiab] OR fistul*[tiab] OR "bowel damage"[tiab]) AND (predict*[tiab] OR prognos*[tiab] OR risk[tiab] OR biomarker*[tiab] OR genom*[tiab] OR transcriptom*[tiab] OR proteom*[tiab] OR metabolom*[tiab] OR microbiom*[tiab] OR imaging[tiab] OR radiomic*[tiab] OR elastograph*[tiab] OR "machine learning"[tiab] OR "artificial intelligence"[tiab])

**Embase**

('crohn disease'/exp OR crohn*:ti,ab,kw) AND ('disease progression'/exp OR progress*:ti,ab,kw OR 'disease behavior':ti,ab,kw OR b1:ti,ab,kw OR b2:ti,ab,kw OR b3:ti,ab,kw OR nonstrictur*:ti,ab,kw OR non-penetrat*:ti,ab,kw OR strictur*:ti,ab,kw OR stenos*:ti,ab,kw OR penetrat*:ti,ab,kw OR fistul*:ti,ab,kw OR 'bowel damage':ti,ab,kw) AND ('prognosis'/exp OR 'biological marker'/exp OR predict*:ti,ab,kw OR prognos*:ti,ab,kw OR risk:ti,ab,kw OR biomarker*:ti,ab,kw OR genom*:ti,ab,kw OR transcriptom*:ti,ab,kw OR proteom*:ti,ab,kw OR metabolom*:ti,ab,kw OR microbiom*:ti,ab,kw OR imaging:ti,ab,kw OR radiomic*:ti,ab,kw OR elastograph*:ti,ab,kw OR 'machine learning':ti,ab,kw OR 'artificial intelligence':ti,ab,kw)

**Web of Science Core Collection**

TS=(Crohn* AND (progress* OR "disease behavior" OR B1 OR B2 OR B3 OR nonstrictur* OR "non-strictur*" OR nonpenetrat* OR "non-penetrat*" OR strictur* OR stenos* OR penetrat* OR fistul* OR "bowel damage") AND (predict* OR prognos* OR risk OR biomarker* OR genom* OR transcriptom* OR proteom* OR metabolom* OR microbiom* OR imaging OR radiomic* OR elastograph* OR "machine learning" OR "artificial intelligence"))

**Supplementary search and selection procedures**

Backward and forward citation tracking of key longitudinal cohorts, prediction-model studies, guidelines, consensus statements, and prior reviews supplemented the database searches. Eligible evidence comprised peer-reviewed full-text Crohn disease studies addressing longitudinal behavioral progression, structural damage, prognostic biomarkers, prediction models, or mechanisms relevant to candidate-feature selection. Direct progression evidence required temporal precedence of the predictor and a baseline phenotype permitting assessment of incident B2 and/or source-defined B3 progression. The term internal B3 was used only when internal penetrating events were explicitly distinguished from perianal disease in the source study. Mixed-baseline or related-outcome studies were retained as indirect prognostic evidence, and established-disease, resection-tissue, organoid, or experimental studies were used for biological interpretation. Preprints, conference abstracts, submitted manuscripts, personal communications, and reports without a relevant Crohn disease progression, prognostic, or mechanistic question were excluded.

Screening followed a single-reviewer narrative workflow. Records were assessed for topical relevance and, when needed, full-text eligibility, then classified by baseline phenotype, predictor–outcome temporality, endpoint definition, and role in the synthesis. Because the original iterative searches did not prospectively retain a deduplicated record log, exact initial retrieval, duplicate-removal, and exclusion counts are unavailable. Supplementary Tables S1–S5 document 52 unique reports used in the detailed evidence map. For descriptive report-level counting, each report was assigned once to its primary evidence-directness tier using baseline phenotype, predictor-outcome temporality, and endpoint definition; where a report could reasonably span two levels, the less-direct tier was used. This yielded Tier 1, n = 3; Tier 2, n = 26; and Tier 3, n = 23. Additional guidelines, reviews, and contextual sources are cited in the main manuscript.
